# An exploration of methods for obtaining 0 = dead anchors for latent scale EQ-5D-Y values

**DOI:** 10.1007/s10198-020-01205-9

**Published:** 2020-06-06

**Authors:** Koonal K. Shah, Juan Manuel Ramos-Goñi, Simone Kreimeier, Nancy J. Devlin

**Affiliations:** 1 PHMR, London, UK; 2 School of Health and Related Research, University of Sheffield, Sheffield, UK; 3 Office of Health Economics, London, UK; 4 Axentiva Solutions, Tacoronte, Spain; 5 Office of the EuroQol Research Foundation, Rotterdam, Netherlands; 6 School of Public Health, Bielefeld University, Bielefeld, Germany; 7 School of Population and Global Health, University of Melbourne, Melbourne, Australia

**Keywords:** EQ-5D-Y, Children, Valuation, Stated preferences, Quality-adjusted life year, I10 – Health, General

## Abstract

**Objectives:**

Discrete choice experiments (DCEs) can be used to obtain latent scale values for the EQ-5D-Y, but these require anchoring at 0 = dead to meet the conventions of quality-adjusted life year (QALY) estimation. The primary aim of this study is to compare four preference elicitation methods for obtaining anchors for latent scale EQ-5D-Y values.

**Methods:**

Four methods were tested: visual analogue scale (VAS), DCE (with a duration attribute), lag-time time trade-off (TTO) and the location-of-dead (LOD) approach. In computer-assisted personal interviews, UK general public respondents valued EQ-5D-3L health states from an adult perspective and EQ-5D-Y health states from a 10-year-old child perspective. Respondents completed valuation tasks using all four methods, under both perspectives.

**Results:**

349 interviews were conducted. Overall, respondents gave lower values under the adult perspective compared to the child perspective, with some variation across methods. The mean TTO value for the worst health state (33333) was about equal to dead in the child perspective and worse than dead in the adult perspective. The mean VAS rescaled value for 33333 was also higher in the child perspective. The DCE produced positive child perspective values and negative adult perspective values, though the models were not consistent. The LOD median rescaled value for 33333 was negative under both perspectives and higher in the child perspective.

**Discussion:**

There was broad agreement across methods. Potential criteria for selecting a preferred anchoring method are presented. We conclude by discussing the decision-making circumstances under which utilities and QALY estimates for children and adults need to be commensurate to achieve allocative efficiency.

**Electronic supplementary material:**

The online version of this article (10.1007/s10198-020-01205-9) contains supplementary material, which is available to authorized users.

## Introduction

The EQ-5D-Y (Youth; three-level version^1^) has been developed as a measure of health outcomes suitable for children and adolescents [1, 2]. However, no value sets are currently available, so EQ-5D-Y data cannot currently be used to estimate quality-adjusted life years (QALYs), as required for cost-utility analysis. The EuroQol Group has recognised the need to establish a protocol for conducting EQ-5D-Y valuation studies.

Two methodological EQ-5D-Y valuation studies undertaken to date—one using visual analogue scale (VAS) [3] and the other using composite time trade-off (C-TTO) and a discrete choice experiment (DCE) with death [4]—have reported somewhat contradictory results. Both studies reported differences in values elicited under adult health and child health perspectives (i.e. from respondents’ own perspective and imagining the health states from the perspective of a child, respectively), but in different directions: Kind et al. reported lower mean VAS ratings for the child perspective compared to the adult perspective, while Kreimeier et al. reported higher mean TTO values for the child perspective. The higher TTO values for the child perspective might have been driven by respondents’ aversion or unwillingness to trade off life years for a child (i.e. to choose to effectively shorten a child’s life). Both of the valuation techniques used by Kreimeier et al. included direct comparisons of health states with (immediate) death, whereas the VAS approach used by Kind et al. did not include any attempt to compare with or anchor at dead. Evidence from Kreimeier et al. suggests that relative preferences regarding dimensions/levels are different for the EQ-5D-3L elicited under the adult perspective and the EQ-5D-Y elicited under the child perspective. However, the authors did not find statistically significant differences across perspectives in the valuation of health state 33333 (the worst state in both the EQ-5D-3L and EQ-5D-Y descriptive systems). The Kind et al. study did not include health state 33333 in its design.

The ‘standard’ DCE (as opposed to DCE plus duration/death) seems to be a feasible solution for eliciting preferences under a child perspective as no time is attached to the health states, thus avoiding the issues raised by asking respondents to sacrifice the duration of a child’s life. Indeed, such preference data for the EQ-5D-Y have been collected from a sample of the UK general population, and are reported elsewhere [5, 6]. However, the DCE-estimated utilities based on those relative preferences are on an undefined scale, which cannot be used directly in QALY calculations [7]. Latent scale DCE data require an anchor point that must be obtained from an additional task or method.

Based on the evidence described above, a key question remains: if we are to use DCE for valuing EQ-5D-Y health states, what is the appropriate method for anchoring the resulting latent scale values? This study tests and compares four methods:

- Visual analogue scale (VAS).
- Lag-time TTO.
- Discrete choice experiment with duration (DCEd; described elsewhere as DCE_TTO_ [8]).
- Location-of-dead (LOD) method, part of the personal utility function (PUF) approach.

The aims of the study are: to explore the use of these four alternative methods for establishing anchors and the resulting values for health state 33333; to compare anchors for the EQ-5D-3L/adult perspective and the EQ-5D-Y/child perspective; and to inform the development of a protocol for valuing the EQ-5D-Y.

## Methods

### Instruments

We used two versions of the EQ-5D instrument: the EQ-5D-3L [9] to describe adult health states and the EQ-5D-Y [10] to describe child health states. Both instruments comprise broadly the same five dimensions with three levels of response, usually coded 1, 2 and 3, producing health states that can be summarised using five-digit codes (profiles)—e.g. 11111 represents no problems in any dimension; 33333 represents the worst possible health state in either descriptive system. However, the instruments differ in wording. The EQ-5D-3L uses wording considered appropriate for adults, while the EQ-5D-Y was developed as an adaptation of the EQ-5D-3L for use in child and adolescent populations, with changes made to the labels for various dimension and level descriptions. For example, the ‘self-care’ and ‘anxiety/depression’ dimensions are re-labelled as ‘looking after myself’ and ‘feeling worried, sad or unhappy’ in the EQ-5D-Y (to avoid confusion, we use the adult labels throughout this manuscript). Further, three of the five level 3 descriptors in the EQ-5D-Y describe having ‘a lot of problems’ with the relevant health dimension. This contrasts with the EQ-5D-3L which refers to being ‘confined to bed’ or ‘unable to [wash or dress myself/perform my usual activities]’.

### Valuation techniques

There exists a broad range of valuation techniques that produce values on a scale anchored at 0 (dead) and 1 (full health). In this study, we focused on the four described below. The first three are widely used by health preference researchers [11, 12]. TTO and DCE are the methods currently favoured for the valuation of the EQ-5D-5L instrument [7], albeit different variants of those methods (composite TTO and DCE without duration, respectively) compared to the variants used in this study. VAS is a relatively simple, non-choice-based method, generally agreed to represent the most feasible of the various valuation techniques [12]. The fourth method—LOD—is a novel technique [13] considered promising by the authors for the purpose of establishing the location of the dead within a descriptive system.

These methods permit latent scale DCE data to be anchored using the value obtained for health state 33333. Other anchoring methods, such as mapping DCE values onto TTO, and combining DCE and TTO data in a hybrid model, have been examined elsewhere [14].

### VAS

The VAS exercise involves rating health states (lasting for 10 years, followed by death) or descriptors on a 0-to-100 scale (ranging from ‘The worst health you can imagine’ to ‘The best health you can imagine’). If ratings for ‘Dead’ and ‘11111’ are obtained, then the rating for health state h can be rescaled using the formula: (Rating_h_ − Rating_dead_)/(Rating_11111_ − Rating_dead_). The rescaled rating is upper bounded at 1 and anchored at 0 = dead.

### TTO

We used the lag-time variant of TTO [15, 16]. The lag-time TTO involves, as its starting point, a choice between 20 years in full health followed by death (life A) and 10 years in the EQ-5D health state under evaluation, followed by 10 years in full health (the ‘lag-time’), followed by death (option B). Respondents could indicate that they preferred life A, preferred life B, or considered both lives to be ‘about the same’. Depending on their choice, the amount of time in full health in life A was varied using the same iterative approach as used in the current EQ-5D-5L valuation protocol [17]. The task ended when the respondent indicated that life A and life B are about the same. The value for the health state could be calculated (assuming zero temporal discounting) as follows: *U* = (*t *− 10)/10, where *U* is the value (utility) and t is the number of years in full health in life A at the respondent’s point of indifference.

Lag-time TTO was used in favour of lead-time TTO (as used by Kreimeier et al. [4] for the valuation of worse-than-dead health states) because in the former the health state under evaluation occurs at the start of the time frame—i.e. if the scenario were to apply to a 10-year-old child, the health state would be experienced whilst the individual in question is still in childhood. However, in lead-time TTO the health state being evaluated occurs after 10 years of full health—i.e. the health state would not be experienced until adulthood. It is acknowledged that if a 10-year-old child enters a health state which then lasts for 10 years, then part of their time experiencing the health state would be in adulthood (particularly given that the EQ-5D-Y is designed for use in 8-to-15-year olds). However, it was deemed useful to maintain consistency with previous EQ-5D-Y valuation work, which had used standard 10-year timeframes [4].

### DCEd

The DCEd exercise comprised a series of forced-choice paired comparisons. Respondents were asked to choose which they preferred out of two EQ-5D health states, each lasting a specified duration (1, 3, 6 or 10 years), followed by death. No indifference option was available.

### LOD

The LOD exercise, developed as part of the PUF approach, seeks to locate each respondent’s position of the dead within a descriptive system. It is a simplified version of the approach used by Devlin et al. [13] and comprised two parts. First, a ranking task was presented requiring respondents to rank level 1 descriptors for each of the EQ-5D dimensions (e.g. ‘no pain or discomfort’) from ‘most important’ to ‘least important’, thereby asking respondents to consider on which dimensions it was most important to avoid problems. Ties were not permitted. Second, a series of forced-choice paired comparison tasks were presented, each involving a choice between living in a specified EQ-5D health state lasting 10 years (followed by death) and 0 years of life (i.e. immediate death). The information gathered in the ranking task was used to personalise the selection of the health states presented in the paired comparison tasks. This was done via a simple algorithm that applied a rating of 100 to the highest-ranked dimension and progressively smaller ratings to the second, third, fourth and bottom-ranked dimensions. Each rating was then weighted by 1, 0.5 or 0 depending on whether they applied to levels 1, 2 or 3 for the relevant dimension. The weighted ratings were summed to generate a total score for each of the 243 possible health states, thereby allowing a personalised ranking of those health states. The paired comparison tasks were designed to identify the individual’s dividing line between states considered to be better or worse than dead. Hence, the ranking task responses played an indirect role in determining the anchor points using the LOD method.

### Study design

All respondents completed all valuation tasks using two different perspectives. In the adult perspective, they were asked to consider their own health, with the EQ-5D-3L instrument used to describe the health states. In the child perspective, they were asked to consider the health of a 10-year-old child, with the EQ-5D-Y instrument used to describe the health states, following the approach used in previous research [4, 5]. No specific instruction was provided about the identity of the 10-year-old child. Half of the respondents were randomly allocated to completing the tasks for the adult perspective first; the other half completed the tasks for the child perspective first. At the half-way point, a pop-up message appeared on the screen advising respondents of the change in perspective. Interviewers were also instructed to advise respondents of this change.

**Fig. 1:**
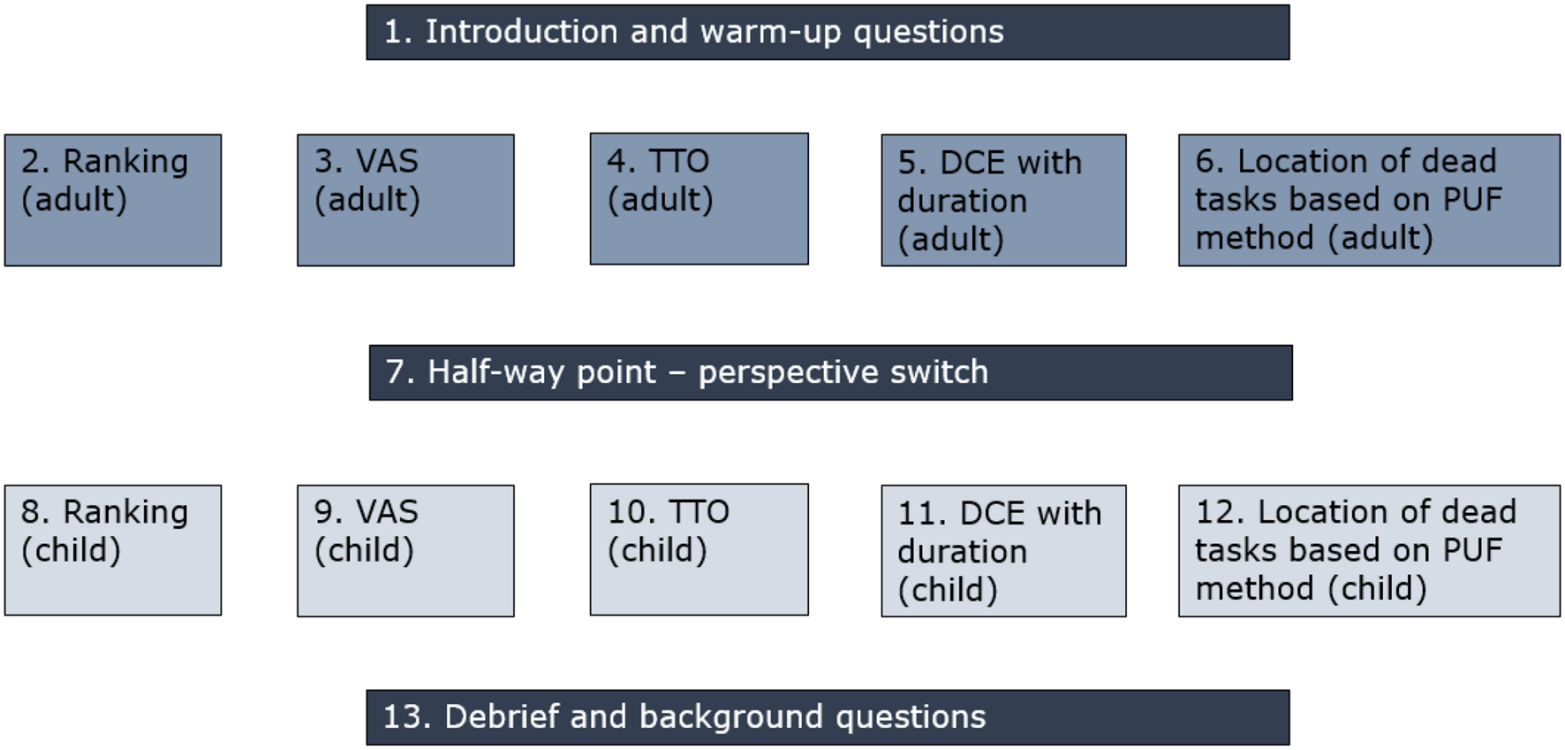
Ordering of the tasks for respondents randomised to the ‘adult perspective first’ arm

The survey design (Fig. 1) was developed with the view to minimising respondent burden: given the relatively large number of valuation techniques and perspectives being used, we opted to minimise the numbers of tasks for each valuation technique:

1. Ranking—single task involving ranking of EQ-5D level 1 descriptors (as needed for the LOD technique).
2. VAS—ratings for 33333 and Dead. With these two ratings, and assuming that the rating for 11111 is 100 (assumption not tested as part of this study), we were able to calculate an anchored value for 33333.
3. Lag-time TTO—valuations for 22,222 (as a warm-up task) and 33333. Note that the TTO technique produced values on the 0 and 1 anchored scale.
4. DCEd—this technique does not produce values directly. Values were estimated by modelling; therefore, a specific experimental design was needed. We used a six-step approach. First, we prepared the set of all 2430 possible candidates with an overlap in two dimensions, no dominant pairs and no repetitions. Second, we simulated 2000 designs each including 42 pairs. Using the D-efficiency measure based on a main effects model, we extracted all pairs included in the best 20 designs. Third, based on priors from Rivero-Arias et al. [5] we estimated the choice probabilities for the pairs from step 2. Fourth, using these estimated probabilities, we divided those pairs into three categories: (a) *P* ≤  0.2; (b) 0.2 < *P* ≤  0.35; and (c) 0.35 < *P* ≤  0.5 (same for *P* > 0.5 applies to B state). For (a) we used the high distance between durations of each pair (i.e. 1 year in one state versus 10 years in the other) with the longer duration for the less likely state. For (b), we used a small distance between durations of each pair and the longer duration is for the less likely state. For (c), we used all possible combinations of durations (1, 3, 6, 10 years). Fifth, based on the Bansback et al. model [18], where the time was an interaction, we simulated 2000 designs with all possible pairs and selected the best based on the D-efficiency measure. Finally, we blocked the design into six blocks (thereby allocating seven DCE pairs to be completed by each respondent) by minimising the variance of the level balance between blocks. We used the same design for both perspectives.
5. LOD—this technique does not produce values directly. Respondents were asked to complete up to five paired comparison tasks, each involving a choice between 10 years in a specified health state followed by death (option A) and 0 years/immediate death (option B). No indifference option was available. The health states presented were selected based on a simple algorithm that used each respondent’s responses to the earlier ranking task to generate a personalised ranking of all 243 health states—see above. The algorithm assumed an equal distance (in utility terms) between each dimension rank (i.e. the difference between the first- and second-ranked dimensions was deemed equal to the difference between the second- and third-ranked dimensions), and between levels (i.e. the difference between level 3 and level 2 was deemed equal to the difference between level 2 and level 1). A random number function was used to break ties to generate the ranking. The health state presented in the first task was always 33333 (ranked 243rd for all respondents). Respondents choosing 33333 over immediate death were not given further choice tasks but were asked if they could think of any health problems that were so bad that they would rather choose immediate death, and if so, to describe those problems using an open-ended text box. Respondents choosing immediate death over 33333 proceeded to a second task in which 33333 was replaced by the health state ranked 122nd (half-way between 1st and 243rd; this health state varied from respondent to respondent). In the subsequent tasks, the health state presented either improved or worsened in ranking/estimated personal utility depending on the respondent’s choice in the previous task. An iterative bisection procedure was used for this purpose [19]. Following the fifth task, each respondent’s location of dead could be estimated to be within a range comprising 15–16 health states.

The adult perspective and EQ-5D-3L were used since the aim of the study was to compare anchor points across instruments. However, a small number of additional interviews (*n* = 50), using an otherwise identical survey design, were conducted with respondents valuing EQ-5D-Y health states throughout, in both the adult and the child perspectives. This allowed a comparison of the data collected using different perspectives whilst controlling for the descriptive system. Results relating to this ‘extended sample’ are provided in the supplementary appendix.

The valuation tasks were preceded by a small number of warm-up and background questions and followed by debrief and further background questions.

### Data collection

Data were collected from members of the UK general population. The survey was administered via the EuroQol Group Valuation Technology (EQ-VT) platform. The EQ-VT was used as the basis for computer-assisted, one-to-one personal interviews in the homes of respondents, undertaken by a team of five experienced interviewers. The interviewers completed a 1-day training session on the methodology and procedures for this study and were asked to follow step-by-step instructions and a script to minimise interviewer bias.

The main data collection was preceded by a pilot, which comprised nine cognitive interviews. In addition to completing the valuation tasks using the adapted EQ-VT, pilot respondents were asked probing questions about how they interpreted the tasks, what they found difficult, and how the questionnaire could be improved. All the cognitive interviews were undertaken by two moderators with expertise in qualitative research methods and were carried out in the offices of the moderators’ employer. The cognitive interviews were audio recorded and transcribed. Some minor improvements were made to the software (e.g. amendment of on-screen explanatory text) based on the findings of the pilot.

An adapted version of the quality control process developed for EQ-5D-5L valuation studies [20] was followed to ensure protocol compliance. Ethics approval for the survey and data collection procedures was granted by the Ethics Committee of the University of Sheffield’s School of Health and Related Research (approval reference: 011675).

### Sample

Sample size calculations were based on requirements to estimate DCEd models. We estimated that a minimum of 300 (50 × 6) respondents would be needed assuming a requirement of about 50 observations for each of the six blocks of pairs included in the DCEd design. We took the average of two rule of thumb recommendations—by Lancsar and Louviere [21] (minimum 20 observations per pair) and Hensher et al. [22] (minimum 30 observations per pair)—and doubled that average to be conservative. The sample comprised adult members of the general population (aged 18 years and older) in two regions in the UK (Midlands and London/Southeast). The sample was recruited using a ‘door knock’ approach, with interviewers approaching a household member of every third home in a randomly allocated postal area and scheduling interview appointments for those individuals that agreed to participate. A recruitment questionnaire was used to ensure that the sample was broadly representative of the general population in terms of age and gender. Respondents received a shopping voucher worth GBP £10 to thank them for their participation.

The sample for the pilot comprised adult members of the general population in London, recruited using a mixed on-street and ‘door knock’ approach. Pilot respondents received a shopping voucher worth GBP £40 to thank them for their participation.

### Analysis

Sample background characteristics were described using frequencies and percentages. Box plots were used for describing and comparing lag-time TTO and rescaled VAS values for 33333. TTO values observed at 0 and − 1 were not treated as censored. The DCEd data were described using observed choice probabilities for each of the pairs included in the design. DCEd values for 33333 were calculated via different models, including the regular conditional logit model, and conditional logit models assuming non-constant proportionality [23]. We estimated models assuming a fixed ½ power and allowing the model to estimate the best-fitted power. Further details of the modelling can be found in Table A3 of the Supplementary Appendix.

Each respondent’s set of choices in the LOD tasks resulted in a range of states within which dead was deduced to be located (for example, for respondents who chose option A in the first task and option B in all subsequent tasks, it was deduced that they located dead between the 228th and the 243rd health states within their own personal ranking). This approach was not possible for respondents who chose option B in the first LOD task, implying that they located dead below 33333 and, therefore, beyond the descriptive system. For each of the 16 deduced regions, the midpoint rank of the range was calculated and the latent utility corresponding to that midpoint was estimated based on the mixed logit model results from the EQ-5D-Y latent scale DCE study reported by Rivero-Arias et al. [5]. This was done by summing the Rivero-Arias et al. coefficients/disutilities for the relevant dimension-levels for each of the 243 health states. That study produced latent utilities based on the DCE responses of a different sample from the present study (albeit also a representative sample of the UK general public), so combining the data in this way relies on an assumption that respondents in the present study would have responded in the same way as respondents in the Rivero-Arias et al. study had they completed a similar DCE survey. These latent utilities ranged from 0 (corresponding to 11111) to − 9.306 (corresponding to 33333; i.e. sum of the five level 3 coefficients/disutilities reported by Rivero-Arias et al.). The value for 33333 was then rescaled onto the 0 (dead) and 1 (full health) scale using the formula: rescaled_33333_ = (latent_33333 _− latent_dead_)/(latent_11111 _− latent_dead_).

Analyses were undertaken using Microsoft Excel and Stata software.

## Results

The main interviews were conducted between September and December 2017. The sample comprised 299 respondents; a further respondent found the subject matter distressing during the interview and asked to withdraw from the study. No respondents who completed their interview in full were excluded. The mean (median) duration of the interview was 40.0 (39.1) minutes. The sample was broadly representative of the general population in terms of age and gender [24], though the oldest individuals (aged 70 years and over) are slightly underrepresented (Table 1). The majority of the respondents are parents, though in many cases their children are now adults.

**Table 1:** Sample background characteristics

	Sample		Population
	*n*	%	%
Age			
18–29	58	19.4	20.0
30–39	55	18.4	16.8
40–49	44	14.7	17.1
50–59	60	20.1	16.7
60–69	45	15.1	13.7
70+	37	12.4	15.8
Gender			
Female	151	50.5	51.1
Male	148	49.5	48.9
Experience of serious illness			
In self	69	23.1	N/A
In family	190	63.5	N/A
In caring for others	77	25.8	N/A
Self-reported EQ-5D profile			
11111	184	62.5	N/A
Any other health state	112	37.5	N/A
Children			
No children	66	22.1	N/A
Youngest child is < 11 years	84	28.1	N/A
Youngest child is 11–18 years	25	8.4	N/A
Youngest child is > 18 years	124	41.5	N/A
Experience of working with children			
Yes	60	20.1	N/A
No	239	79.9	N/A

### Ranking

Anxiety/depression was the highest-ranked (considered the most important) dimension on average (i.e. based on mean rank) in the child perspective but only the third-highest ranked in the adult perspective. In the adult perspective, usual activities was the highest-ranked dimension; this was the third-highest ranked in the child perspective. Mobility was found to be the lowest-ranked (least important) dimension on average under both perspectives.

### VAS

**Fig. 2:**
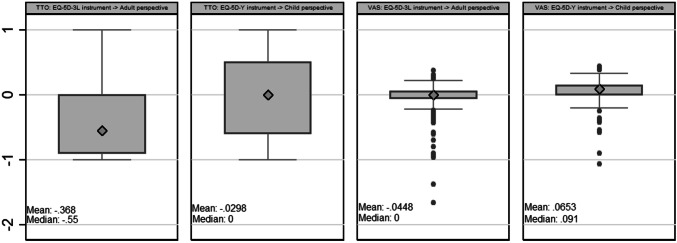
Box-plots of TTO and rescaled VAS values for health state 33333^a^. ^a^One outlier VAS value lower than − 3 was removed from the graph for scaling purposes

On average, VAS ratings and values (rescaled ratings) given to 33333 were higher in the child perspective than in the adult perspective (Fig. 2). A clear majority of respondents considered 33333 to be better than dead when answering from a child perspective; whereas under the adult perspective the most common response was to rate 33333 as worse than dead.

### TTO

The average value given to 33333 in the child perspective was close to 0 (or, taking the median, exactly 0), whereas in the adult perspective the average value was clearly negative. The majority of respondents gave a higher value to 33333 in the child perspective than in the adult perspective (Fig. 2). Four of the 349 respondents (1.1%) gave a lower value to 22,222 than to 33333. Excluding these ‘inconsistent’ respondents lowered the mean value for 33333 by 0.006 in the child perspective, while the corresponding difference in the adult perspective was even smaller (0.003).

### DCEd

DCEd model results were in line with VAS and TTO results to the extent that values for 33333 were negative for the adult perspective and positive for the child perspective (this result was consistent across all models). Observed choice probabilities showed a preference for longer life duration in the child perspective (Table 2). This preference for longer duration meant that models were not consistent (i.e. some logically worse health states have higher utilities than logically better, or dominant, health states) in the child perspective. It seems that respondents focused more on the duration of the lives than to the health problems described. The DCEd results indicate that respondents generally avoided shorter life durations and problems with pain/discomfort when considering the health of a 10-year-old child, whereas they focused on problems with mobility and pain/discomfort when considering their own (adult) health.

**Table 2:** Discrete choice experiment with duration observed choice probabilities

Health state 1	Years in health state 1	Health state 2	Years in health state 2	EQ-5D-3L —> Adult perspective vs EQ-5D-Y —> Child perspective			EQ-5D-Y —> Adult perspective vs EQ-5D-Y —> Child perspective		
				Adult perspective	Child perspective	Diff adult–child	Adult perspective	Child perspective	Diff adult–child
11321	10	31211	1	0.633	0.653	− 0.020	0.250	0.250	0.000
11321	3	31211	6	0.479	0.313	0.167	0.333	0.333	0.000
11322	6	12221	1	0.540	0.540	0.000	0.500	0.500	0.000
11323	3	31222	1	0.563	0.604	− 0.042	0.500	0.667	0.167
12112	1	11213	10	0.438	0.333	0.104	0.500	0.417	− 0.083
12122	10	31112	1	0.569	0.549	0.020	0.500	0.500	0.000
12211	3	11222	6	0.404	0.447	− 0.043	0.625	0.625	0.000
12313	10	13111	1	0.447	0.553	− 0.106	0.375	0.250	− 0.125
12322	6	32221	3	0.596	0.617	− 0.021	0.500	0.250	− 0.250
13113	10	22112	1	0.633	0.653	− 0.020	0.250	0.625	0.375
13233	10	33113	3	0.588	0.510	0.078	0.667	0.333	− 0.333
13331	10	23211	3	0.451	0.510	− 0.059	0.333	0.167	− 0.167
13332	10	22322	3	0.426	0.574	− 0.149	0.500	0.250	− 0.250
13332	6	32312	1	0.537	0.519	0.019	0.625	0.625	0.000
21133	10	22122	1	0.500	0.521	− 0.021	0.500	0.500	0.000
21223	6	31211	3	0.537	0.537	0.000	0.625	0.250	− 0.375
21233	10	21322	1	0.556	0.481	0.074	0.625	0.500	− 0.125
21322	6	31311	10	0.480	0.46	0.020	0.500	0.375	− 0.125
22233	6	31133	10	0.429	0.388	0.041	0.500	0.500	0.000
22323	10	31321	6	0.520	0.500	0.020	0.375	0.500	0.125
22332	10	23311	3	0.438	0.396	0.042	0.250	0.500	0.250
22333	10	23132	3	0.519	0.519	0.000	0.500	0.500	0.000
23111	1	13331	10	0.490	0.408	0.082	0.500	0.250	− 0.250
23213	10	31211	6	0.551	0.633	− 0.082	0.375	0.500	0.125
23223	6	32123	10	0.611	0.407	0.204	0.375	0.500	0.125
23312	1	31311	6	0.468	0.404	0.064	0.750	0.500	− 0.250
23321	1	22333	6	0.553	0.426	0.128	0.500	0.625	0.125
31111	10	21212	3	0.520	0.500	0.020	0.500	0.500	0.000
31111	1	21123	6	0.375	0.354	0.021	0.583	0.333	− 0.250
31111	3	12112	10	0.333	0.313	0.021	0.333	0.250	− 0.083
31111	6	11312	10	0.388	0.327	0.061	0.500	0.625	0.125
31231	10	33111	3	0.556	0.481	0.074	0.625	0.625	0.000
31233	10	32221	1	0.400	0.540	− 0.140	0.625	0.500	− 0.125
31323	10	32122	3	0.420	0.480	− 0.060	0.375	0.375	0.000
32111	6	23311	10	0.389	0.370	0.019	0.625	0.500	− 0.125
32133	1	13233	10	0.431	0.471	− 0.039	0.167	0.500	0.333
32211	3	13212	10	0.383	0.404	− 0.021	0.500	0.625	0.125
33122	1	23332	10	0.431	0.412	0.020	0.500	0.667	0.167
33211	3	33132	10	0.520	0.480	0.040	0.375	0.375	0.000
33212	1	23233	10	0.490	0.408	0.082	0.500	0.250	− 0.250
33212	3	13223	6	0.392	0.412	− 0.020	0.500	0.500	0.000
33212	6	23223	10	0.451	0.412	0.039	0.500	0.333	− 0.167
Predicted values for 33333									
Logit model				− 0.796	0.059				
Power model (power = 1/2)				− 0.468	0.280				
Power model (power = 0.296)				− 0.227	0.188				

Models coefficients are reported in the Appendix (Table A1)

### LOD

One respondent (0.3%) chose option B in all of the LOD tasks, implying that all of the health states presented were worse than dead. Conversely, a sizeable minority of respondents chose option A in the first task, implying that 33333 is better than dead. The proportion of respondents making this choice was higher in the child perspective (32.8%) than in the adult perspective (23.1%). When asked if they could think of any health states that were so bad that they would rather choose immediate death, 57.0% of the respondents in the child perspective and 53.6% of respondents in the adult perspective said that they could. Most of the descriptions of these ‘worse than dead’ states—in both the child and adult perspectives—focused on being in vegetative states and/or having severe brain damage.

Overall, dead was located lower in the descriptive system in the child perspective than in the adult perspective, resulting in higher rescaled values (Table 3)—in other words, respondents located dead amongst more severe health states in the child perspective. The mean rescaled values shown in Table 3 underestimate the actual value for 33333, since they do not take into account the fact that for respondents who chose option A in the first task, the rescaled value for 33333 should be positive. Including such positive values would have an upward effect on the mean; it is worth noting that this effect would be stronger in the child perspective since more respondents chose option A in the first task in this version. The median rescaled values are unaffected by this issue since the median respondent chose option B on at least one occasion.

**Table 3:** Summary of LOD results

Set of choices	Deduced range in which dead is located	Midpoint of deduced range (rank)	Latent utility of midpoint	Rescaled utility for 33333				
					Adult perspective		Child perspective	
					*n*	%	*n*	%
BBBBB	1st to 17th ranked states	9	− 1.015	− 8.170	1	0.3	0	0.0
BBBBA	17th to 32nd ranked states	24.5	− 1.826	− 4.098	6	2.0	0	0.0
BBBAB	32nd to 47th ranked states	39.5	− 2.290	− 3.064	4	1.3	1	0.3
BBBAA	47th to 62nd ranked states	54.5	− 2.690	− 2.459	16	5.4	9	3.0
BBABB	62nd to 77th ranked states	69.5	− 3.048	− 2.053	6	2.0	1	0.3
BBABA	77th to 92nd ranked states	84.5	− 3.415	− 1.725	15	5.0	13	4.3
BBAAB	92nd to 107th ranked states	99.5	− 3.728	− 1.496	9	3.0	5	1.7
BBAAA	107th to 122nd ranked states	114.5	− 4.033	− 1.307	25	8.4	19	6.4
BABBB	122nd to 138th ranked states	130	− 4.399	− 1.116	11	3.7	4	1.3
BABBA	138th to 153rd ranked states	145.5	− 4.717	− 0.973	9	3.0	11	3.7
BABAB	153rd to 168th ranked states	160.5	− 5.005	− 0.859	11	3.7	14	4.7
BABAA	168th to 183rd ranked states	175.5	− 5.383	− 0.729	18	6.0	18	6.0
BAABB	183rd to 198th ranked states	190.5	− 5.776	− 0.611	17	5.7	14	4.7
BAABA	198th to 213th ranked states	205.5	− 6.218	− 0.497	18	6.0	21	7.0
BAAAB	213th to 228th ranked states	220.5	− 6.822	− 0.364	21	7.0	20	6.7
BAAAA	228th to 243rd ranked states	235.5	− 7.825	− 0.189	43	14.4	51	17.1
A	Dead cannot be located using LOD tasks	N/A	N/A	N/A	69	23.1	98	32.8
Mean rescaled utility for 33333 (excluding respondents who considered 33333 to be better than dead)					− 1.076		− 0.787	
Mean rescaled utility for 33333 (assuming a rescaled utility of 0 for respondents who considered 33333 to be better than dead)					− 0.828		− 0.529	
Median rescaled utility for 33333					− 0.497		− 0.364	

### Comparison across methods

It is possible to report whether each individual respondent valued 33333 as better than dead via the TTO, VAS and LOD tasks (Table 4). Respondents were more likely to value 33333 as better than dead in the child perspective than in the adult perspective. This finding was consistent across all three methods. Respondents valued 33333 as better than dead via VAS more frequently than via the other two methods. The majority of respondents did not provide internally consistent valuations, in that they valued 33333 as better than dead via one of the methods but as worse than or equal to dead via another of the methods.

**Table 4:** Comparison across methods: valuation of 33333 in relation to 0 = dead

	Child perspective		Adult perspective		Both perspectives	
	*n*	%	*n*	%	*n*	%
TTO—respondents valuing 33333 as better than dead	125	41.8	68	22.7	59	19.7
VAS—respondents valuing 33333 as better than dead	210	70.2	110	36.8	96	32.1
LOD—respondents valuing 33333 as better than dead	98	32.8	69	23.1	65	21.7
All three methods—respondents providing internally consistent valuations^a^	109	36.5	159	53.2	70	23.4

^a^i.e. 33333 valued as better than dead using all three methods OR 33333 valued as worse than or equal to dead across all three methods

### Debrief questions

The majority of respondents (81.9%) found the child perspective questions more difficult, with a slight majority (54.5%) claiming that they found it somewhat or very difficult to imagine the health of a 10-year-old child (Table 5). Respondents were varied in terms of what sort of child they were thinking of; the most common approach was to think of ‘no particular child’. The vast majority of respondents (81.6%) claimed that their responses might have been different if they had been asked to consider a child of different age, though no information is available about how their responses would have differed. The majority of respondents (62.9%) indicated that the health system should give equal priority to the treatment of adults and children.

**Table 5:** Responses to debrief questions

Question/response options	*n*	%
Which questions did you find more difficult—the questions about your own health or the questions about the health of a 10-year-old child?		
The questions about my own health were more difficult	10	3.3
The questions about the health of a 10-year-old child were more difficult	245	81.9
Both types of questions were equally difficult	44	14.7
None of the above/don’t know	0	0.0
How easy or difficult did you find it to imagine the health of a 10-year-old child?		
Very easy	18	6.0
Somewhat easy	61	20.4
Neither easy nor difficult	57	19.1
Somewhat difficult	98	32.8
Very difficult	65	21.7
What sort of child were you thinking of when responding to the questions?		
My own child	102	34.1
A child that I know (but not my own child)	46	15.4
No particular child	122	40.8
Myself as a child	11	3.7
None of the above/don’t know	18	6.0
Would your responses to the questions have been different if you had been asked to imagine a child of a different age—for example, a 5 year old child?		
Yes	244	81.6
No	55	18.4
How do you think a health care system with a limited budget should prioritise resources?		
The health system should prioritise the treatment of adults	0	0.0
The health system should prioritise the treatment of children	110	36.8
The health system should give equal priority to the treatment of adults and children	188	62.9
Don’t know	1	0.3

## Discussion

Our findings in this study were that three of the methods we tested are feasible to use to obtain stated preference-based anchors for a potential EQ-5D-Y value set (LOD’s failure to handle cases where 33333 is considered better then dead arguably makes it the least feasible). This opens the possibility that the relative importance of dimensions could be rapidly and inexpensively obtained for EQ-5D-Y via DCE, then subsequently anchored at dead = 0 via a smaller-scale (but more resource-intensive) study applying one of the methods reported here. Indeed, while our study was focused on the valuation of the EQ-5D-Y instrument, it is worth noting that in principle this approach could also be followed for the valuation of adult health states using other instruments.

A strong finding from this study was the broad level of agreement across the four very different methods used to locate the relative position of dead = 0 for adult versus child perspectives. Previous studies of EQ-5D-Y valuation, as noted in the introduction, had found contradictory results for TTO and VAS tasks, with values for child health states being higher or lower than corresponding adult health states depending on the method used. However, it is worth noting that the VAS study reported by Kind et al. [3] did not include 33333 or the rating of dead. Our results are in line with those reported by Kreimeier et al. [4] to the extent that values for 33333 were higher in the child perspective. However, our study found this pattern more clearly in all methods employed.

There are many improvements and alterations that could be made to the specific approaches used to implement all four methods. Notwithstanding that, the evidence from this study suggests that none of the four can be immediately ruled out as being infeasible or not working (though the way in which the LOD data were combined with data from a separate study may be problematic as it requires a high level of agreement between the preferences of the two study samples to be valid). This in turn suggests either that multiple methods could continue to be used in future studies (with conclusions somehow triangulated across methods) or that a choice between them must be made. We have considered the criteria that might be used to guide this choice—our thinking about this is provided here for discussion.

Criteria for choosing between anchoring methods could arguably include:

- Feasibility. We consider multiple methods to be feasible, so in this case feasibility does not identify a single preferred option out of the candidate methods. It should be noted that one respondent in the main study and one respondent in the pilot found the subject matter distressing and their interviews were terminated. This issue does not appear to be linked to any particular valuation technique but rather to the general task of considering the severe ill health and death of children (necessary for all of the candidate methods). Hence, it is worth acknowledging that these kinds of studies are not easy to undertake and can pose a considerable emotional burden on respondents.
- Acceptability to decision makers. This includes any prior beliefs decision makers may have about desirable theoretical properties of methods. For example, NICE requires utilities to be based on ‘choice-based methods’ [25]. TTO and DCEd are generally accepted as being choice-based; the LOD approach is also based on choice-based tasks, though the novelty and relative lack of research using the technique is likely to make it less attractive to decision makers. VAS has tended to be rejected by health economists (with rare exceptions [26]) on the grounds that it is not choice-based.
- Potential for administration on-line. While the current study was undertaken using face-to-face interviews, it may be desirable for future studies to be capable of being completed online. This would probably preclude the lag-time TTO or other TTO variants, because of the complexity of the tasks, but would favour VAS, DCEd and potentially the LOD approach (e.g. as implemented elsewhere [27]).
- Theoretical and empirical coherence with the preference data to be anchored. If unanchored preference data are to be collected via DCE and a second task used for anchoring, it may be considered desirable that there be some degree of consistency or coherence between these two sets of preference data. Our study has proceeded on the basis that this is a legitimate basis for comparing different methods for anchoring the data. VAS valuation may present issues in anchoring latent scale DCE data because the preferences are elicited using completely different sorts of tasks with different biases affecting each. This might favour the use of DCEd—although this raises the question of why DCEd would not then be favoured as the sole approach to eliciting preferences (likewise if TTO emerges as the preferred anchoring method, this raises the question of why TTO would not be used as the sole valuation method rather than obtaining latent scale DCE data that need to be anchored using a second method. Our response to this is that all child health valuation techniques involving duration pose issues, so it is preferable to focus the majority of resources on a non-duration-based approach—i.e. DCE—to obtain as accurate as possible an estimation of the relative importance of different dimensions and levels). In addition, the current state of the art in DCEd, particularly in terms of design and modelling, has yet to achieve a final solution, meaning that further research is needed to understand the dependency of certain kinds of designs on modelling results as we have found in this study. It may also be problematic if the preferences of the sample providing the unanchored data differ systematically from the preferences of the sample providing the data for anchoring purposes. One solution to this would be to use the same sample for both data collection exercises or to ensure that the two samples are matched as closely as possible in terms of observable characteristics.
- Theoretical and empirical consistency with adult valuations in use in HTA. This raises a fundamental consideration: should the values for the EQ-5D-Y, and QALYs estimated from them, be commensurate with those for adult EQ-5D instruments? That is, should a QALY estimated for a child be equal to a QALY estimated for an adult? Where resource allocation decisions are made from a single health care budget, the achievement of allocative efficiency would rely on being able to consider QALYs gained and foregone across both adult and child interventions. Alternatively, if budgets for health care for children are ring-fenced, then the only decisions for which EQ-5D-Y values would be used are to assess the incremental QALY gains and cost-effectiveness of alternative ways of treating children. In the latter case, commensurability with adult values would not be a requirement. So, for example, and given results reported in this paper, the value set for the EQ-5D-Y might contain no states worse than dead. The extent to which budgets and, therefore, cost-effectiveness thresholds, might be characterised as being distinct between adults and children, depends on the nature of the health care system. These normative issues would appropriately be informed by discussions with those responsible for HTA, rather than resting on our judgements as researchers. However, even where the child health care budget is ring-fenced, it is important to note that interventions that avoid the premature death of children involve QALY gains both in childhood and in adulthood, so in practice the complete separation of utilities and QALY estimates is difficult if not impossible.

All four methods used in this paper have their own limitations. The lag-time TTO results relate to a child aged 10 years experiencing health states for 10 years, which takes them to adulthood at 20 years of age, and then experiencing a lag-time period of full health. The time being traded off is, therefore, partly years in young adulthood and (for negative values) partly years in childhood. In addition, a feature of both lead and lag-time TTO is that the minimum value is determined by the ratio of duration in health to lead/lag time (in the current study, − 1) [15]. Further, the amount of lead or lag-time available to trade will affect the distribution of values for severe health states (the more time available, the more time is traded).

Similarly, the LOD approach to locating the position of dead within the descriptive system was, in this study, based on quite limited information about the nature of respondents’ utility functions. Further, there lacks an agreed means of identifying the position of the dead when respondents consider it to be worse than 33333 and, therefore, to lie outside the EQ-5D descriptive system. More sophisticated approaches to this task are possible and can be rendered suitable for use online (e.g. see [27], where a similar approach was embedded within an online adaptive DCE to create an EQ-5D-5L value set for New Zealand).

A further limitation of this study is that anchors for the EQ-5D-Y were obtained by eliciting stated preferences regarding health states pertaining to a child aged 10 years. We judged that specifying the age for the child to be considered in these tasks was important, or else respondents would have introduced their own, varying and unobserved, assumptions about that. Our choice of 10 years of age in this study was influenced by this being the age also used in the UK latent scale DCE study of EQ-5D-Y values [5], which produced the data that we wished to re-scale using the anchors derived in the current study. It is also consistent with previous research by Kind et al. [3] and Kreimeier et al. [4]. Further, 10 years is the mid-point between the ages of 8 and 12 years where the use of EQ-5D-Y is recommended (ages 12–15 being regarded as an area of overlap where EQ-5D-Y is recommended but the adult EQ-5D can also be used) [10]. Nevertheless, the specification of age means that the anchoring results reported here may be specific to that age and might be different for younger or older children. There is some suggestion from our respondents that this is the case, with 83% saying their responses to the tasks might have been different for children of different ages. This is an issue which does not arise in the valuation of adult health states, where respondents are asked to consider health states as if experienced by themselves, at their current age is. However, in both adult and child valuation tasks, there is no guarantee that the preferences obtained and the age of the person imagined to be experiencing the state match the age of the patients reporting EQ-5D-Y data to which those utilities are then applied.

A related limitation is that under the adult perspective, respondents were asked to consider their own health, whereas under the child perspective they were asked to consider the health of another individual. Hence, some of the differences may be due to respondents’ preferences about other individuals rather than about children per se. The importance of differences in perspective when eliciting preferences in health has been examined elsewhere [28–30].

The fact that the majority of respondents did not provide internally consistent valuations across the VAS, TTO and LOD methods is potentially concerning. Further research should focus on the reasons why respondents respond differently to different valuation techniques. Approaches that encourage respondents to ‘think aloud’ and/or to reflect and deliberate on their choices would likely be useful for this kind of research [13, 31].

The decision to include four valuation methods and two perspectives in the study resulted in a rather complex study design (Fig. 1). To minimise respondent burden, the number of tasks included for each method was restricted. This meant that the average interview duration for this study was similar to that for typical EQ-5D-5L valuation studies [32]. However, it may have been beneficial to have included more VAS and TTO health states to assess whether the response patterns observed for 33333 were consistent over the full range.

In conclusion, this study has shown that multiple options exist for providing post-hoc anchors for latent scale DCE preferences. The stated preference methods tested were mostly feasible to use and produced plausible anchors. There was broad agreement between the methods in terms of the placement of the anchor for dead for children versus adults, with the value for 33333 being higher (and more likely to be positive) for children than for adults. The choice between methods, and on what basis that choice should be made, requires further consideration. The choice of anchors raises wider questions about the extent to which the use of values in cost-effectiveness analysis imposes a requirement of commensurability between adult and child health state values.

## Electronic supplementary material

Below is the link to the electronic supplementary material.

Supplementary file1 (DOCX 124 kb)

## Footnotes

1 Sometimes also denoted as EQ-5D-Y-3L, to distinguish it from the newly developed five-level version.
