# Supplementary material for: An exploration of methods for obtaining 0 = dead anchors for latent scale EQ-5D-Y values
Source: Eur J Health Econ. 2020 Jun 6;21(7):1091–103. doi: 10.1007/s10198-020-01205-9 (PMC7423806; doi:10.1007/s10198-020-01205-9)
Supplement: Supplementary file 1 — Supplementary file1 (DOCX 124 kb) [file 10198_2020_1205_MOESM1_ESM.docx]

**Table A1.** Extended sample background characteristics

|  | Extended sample | | Population |
| --- | --- | --- | --- |
|  | n | % | % |
| Age |  |  |  |
| 18-29 | 11 | 22.0% | 20.0% |
| 30-39 | 9 | 18.0% | 16.8% |
| 40-49 | 6 | 12.0% | 17.1% |
| 50-59 | 10 | 20.0% | 16.7% |
| 60-69 | 9 | 18.0% | 13.7% |
| 70+ | 5 | 10.0% | 15.8% |
| Gender |  |  |  |
| Female | 26 | 52.0% | 51.1% |
| Male | 24 | 48.0% | 48.9% |
| Experience of serious illness |  |  |  |
| In self | 4 | 8.0% | N/A |
| In family | 20 | 40.0% | N/A |
| In caring for others | 4 | 8.0% | N/A |
| Self-reported EQ-5D profile |  |  |  |
| 11111 | 37 | 74.0% | N/A |
| Any other health state | 13 | 26.0% | N/A |
| Children |  |  |  |
| No children | 17 | 34.0% | N/A |
| Youngest child is <11yrs | 7 | 14.0% | N/A |
| Youngest child is 11-18yrs | 3 | 6.0% | N/A |
| Youngest child is >18yrs | 23 | 46.0% | N/A |
| Experience of working with children |  |  |  |
| Yes | 4 | 8.0% | N/A |
| No | 46 | 92.0% | N/A |

**Table A2.** DCEd observed choice probabilities for extended sample

| Health state 1 | Years in health state 1 | Health state 2 | Years in health state 2 | Adult  Perspective | Child perspective | Diff  Adult-child |
| --- | --- | --- | --- | --- | --- | --- |
| 11321 | 10 | 31211 | 1 | 0.250 | 0.250 | 0.000 |
| 11321 | 3 | 31211 | 6 | 0.333 | 0.333 | 0.000 |
| 11322 | 6 | 12221 | 1 | 0.500 | 0.500 | 0.000 |
| 11323 | 3 | 31222 | 1 | 0.500 | 0.667 | 0.167 |
| 12112 | 1 | 11213 | 10 | 0.500 | 0.417 | -0.083 |
| 12122 | 10 | 31112 | 1 | 0.500 | 0.500 | 0.000 |
| 12211 | 3 | 11222 | 6 | 0.625 | 0.625 | 0.000 |
| 12313 | 10 | 13111 | 1 | 0.375 | 0.250 | -0.125 |
| 12322 | 6 | 32221 | 3 | 0.500 | 0.250 | -0.250 |
| 13113 | 10 | 22112 | 1 | 0.250 | 0.625 | 0.375 |
| 13233 | 10 | 33113 | 3 | 0.667 | 0.333 | -0.333 |
| 13331 | 10 | 23211 | 3 | 0.333 | 0.167 | -0.167 |
| 13332 | 10 | 22322 | 3 | 0.500 | 0.250 | -0.250 |
| 13332 | 6 | 32312 | 1 | 0.625 | 0.625 | 0.000 |
| 21133 | 10 | 22122 | 1 | 0.500 | 0.500 | 0.000 |
| 21223 | 6 | 31211 | 3 | 0.625 | 0.250 | -0.375 |
| 21233 | 10 | 21322 | 1 | 0.625 | 0.500 | -0.125 |
| 21322 | 6 | 31311 | 10 | 0.500 | 0.375 | -0.125 |
| 22233 | 6 | 31133 | 10 | 0.500 | 0.500 | 0.000 |
| 22323 | 10 | 31321 | 6 | 0.375 | 0.500 | 0.125 |
| 22332 | 10 | 23311 | 3 | 0.250 | 0.500 | 0.250 |
| 22333 | 10 | 23132 | 3 | 0.500 | 0.500 | 0.000 |
| 23111 | 1 | 13331 | 10 | 0.500 | 0.250 | -0.250 |
| 23213 | 10 | 31211 | 6 | 0.375 | 0.500 | 0.125 |
| 23223 | 6 | 32123 | 10 | 0.375 | 0.500 | 0.125 |
| 23312 | 1 | 31311 | 6 | 0.750 | 0.500 | -0.250 |
| 23321 | 1 | 22333 | 6 | 0.500 | 0.625 | 0.125 |
| 31111 | 10 | 21212 | 3 | 0.500 | 0.500 | 0.000 |
| 31111 | 1 | 21123 | 6 | 0.583 | 0.333 | -0.250 |
| 31111 | 3 | 12112 | 10 | 0.333 | 0.250 | -0.083 |
| 31111 | 6 | 11312 | 10 | 0.500 | 0.625 | 0.125 |
| 31231 | 10 | 33111 | 3 | 0.625 | 0.625 | 0.000 |
| 31233 | 10 | 32221 | 1 | 0.625 | 0.500 | -0.125 |
| 31323 | 10 | 32122 | 3 | 0.375 | 0.375 | 0.000 |
| 32111 | 6 | 23311 | 10 | 0.625 | 0.500 | -0.125 |
| 32133 | 1 | 13233 | 10 | 0.167 | 0.500 | 0.333 |
| 32211 | 3 | 13212 | 10 | 0.500 | 0.625 | 0.125 |
| 33122 | 1 | 23332 | 10 | 0.500 | 0.667 | 0.167 |
| 33211 | 3 | 33132 | 10 | 0.375 | 0.375 | 0.000 |
| 33212 | 1 | 23233 | 10 | 0.500 | 0.250 | -0.250 |
| 33212 | 3 | 13223 | 6 | 0.500 | 0.500 | 0.000 |
| 33212 | 6 | 23223 | 10 | 0.500 | 0.333 | -0.167 |

**Table A3.** DCEd model coefficients, using incremental dummies (initial and extended samples)

|  | Adult perspective (All data) | | | | | | Child perspective (All data) | | | | | |
| --- | --- | --- | --- | --- | --- | --- | --- | --- | --- | --- | --- | --- |
|  | Conditional logit | | ½  power | | Best-fitted power | | Conditional logit | | ½  power | | Best-fitted  power | |
|  | Coeff | Stderr | Coeff | Stderr | Coeff | Stderr | Coeff | Stderr | Coeff | Stderr | Coeff | Stderr |
| mo2 | 0.128 | 0.042 | 0.076 | 0.029 | 0.063 | 0.023 | 0.090 | 0.030 | 0.058 | 0.022 | 0.069 | 0.026 |
| mo3 | 0.529 | 0.057 | 0.423 | 0.039 | 0.340 | 0.045 | **-0.052** | 0.052 | **-0.019** | 0.037 | **-0.039** | 0.048 |
| sc2 | 0.087 | 0.055 | 0.081 | 0.034 | 0.073 | 0.025 | **-0.001** | 0.032 | **-0.005** | 0.022 | **-0.009** | 0.026 |
| sc3 | 0.104 | 0.060 | 0.110 | 0.037 | 0.103 | 0.028 | 0.054 | 0.033 | 0.037 | 0.022 | 0.041 | 0.026 |
| ua2 | **-0.094** | 0.053 | 0.010 | 0.030 | 0.040 | 0.021 | **-0.025** | 0.038 | **-0.004** | 0.026 | **-0.019** | 0.034 |
| ua3 | 0.184 | 0.048 | 0.078 | 0.027 | 0.047 | 0.021 | 0.091 | 0.028 | 0.051 | 0.018 | 0.070 | 0.023 |
| pd2 | **-0.169** | 0.081 | 0.012 | 0.045 | 0.060 | 0.029 | 0.056 | 0.045 | 0.024 | 0.027 | 0.031 | 0.033 |
| pd3 | 0.825 | 0.113 | 0.505 | 0.063 | 0.362 | 0.047 | 0.644 | 0.076 | 0.499 | 0.044 | 0.580 | 0.076 |
| ad2 | **-0.101** | 0.055 | **-0.040** | 0.034 | **-0.027** | 0.024 | **-0.031** | 0.038 | **-0.034** | 0.028 | **-0.035** | 0.033 |
| ad3 | 0.304 | 0.048 | 0.215 | 0.028 | 0.165 | 0.024 | 0.116 | 0.028 | 0.114 | 0.018 | 0.123 | 0.022 |
| Cumulative density function | P = exp(A)/  (exp(A) + exp(B)) | | P = A/  (A +B) | | P = A/  (A +B) | | P = exp(A)/  (exp(A) + exp(B)) | | P = A/  (A +B) | | P = A/  (A +B) | |
| Time specification | Value = duration^alpha^ – (mo2 + mo3 + sc2 + ......+ ad2 + ad3) × duration^beta^ | | | | | | | | | | | |
| Power | 1.000  Fixed | | 0.500  Fixed | | 0.304 | 0.053 | 1.000  Fixed | | 0.500  Fixed | | 0.670 | 0.084 |

- Regular domain/levels dummy variables are reported here. All reported as movements from no problems. Time was included as a continuous variable with linear form for the conditional logit model and with a power function in the other two cases.
- Coefficients can be read as disutilities from (1) - full health asociated with each movement from no problems.
- Power function models were estimated as they were selected as the best among different methods, including methods to manage heterogeneity, in a recent DCE modelling competition [21]. The assumption underlying this model is that the duration of the health problems impact on preferences in a non-linear form. Imposing a power relationship means that for a power of 1, it results in the logit model. However, the model fits better when using power values < 1. We therefore first tried 0.5 (like rootsquare) and we also allowed a free estimation.

**Table A4.** Summary of LOD results for extended sample

| Set of  choices | Deduced range in which dead is located | Midpoint of deduced range (rank) | Latent utility of midpoint | Rescaled utility for 33333 | Adult perspective | | Child perspective | |
| --- | --- | --- | --- | --- | --- | --- | --- | --- |
|  |  |  |  |  | n | % | n | % |
| BBBBB | 1st to 17th ranked states | 9 | -1.015 | -8.170 | 0 | 0.0% | 1 | 2.0% |
| BBBBA | 17th to 32nd ranked states | 24.5 | -1.826 | -4.098 | 1 | 2.0% | 1 | 2.0% |
| BBBAB | 32nd to 47th ranked states | 39.5 | -2.290 | -3.064 | 0 | 0.0% | 0 | 0.0% |
| BBBAA | 47th to 62nd ranked states | 54.5 | -2.690 | -2.459 | 3 | 6.0% | 2 | 4.0% |
| BBABB | 62nd to 77th ranked states | 69.5 | -3.048 | -2.053 | 1 | 2.0% | 1 | 2.0% |
| BBABA | 77th to 92nd ranked states | 84.5 | -3.415 | -1.725 | 2 | 4.0% | 2 | 4.0% |
| BBAAB | 92nd to 107th ranked states | 99.5 | -3.728 | -1.496 | 1 | 2.0% | 1 | 2.0% |
| BBAAA | 107th to 122nd ranked states | 114.5 | -4.033 | -1.307 | 2 | 4.0% | 1 | 2.0% |
| BABBB | 122nd to 138th ranked states | 130 | -4.399 | -1.116 | 5 | 10.0% | 2 | 4.0% |
| BABBA | 138th to 153rd ranked states | 145.5 | -4.717 | -0.973 | 4 | 8.0% | 2 | 4.0% |
| BABAB | 153rd to 168th ranked states | 160.5 | -5.005 | -0.859 | 2 | 4.0% | 1 | 2.0% |
| BABAA | 168th to 183rd ranked states | 175.5 | -5.383 | -0.729 | 4 | 8.0% | 1 | 2.0% |
| BAABB | 183rd to 198th ranked states | 190.5 | -5.776 | -0.611 | 1 | 2.0% | 3 | 6.0% |
| BAABA | 198th to 213th ranked states | 205.5 | -6.218 | -0.497 | 4 | 8.0% | 4 | 8.0% |
| BAAAB | 213th to 228th ranked states | 220.5 | -6.822 | -0.364 | 3 | 6.0% | 1 | 2.0% |
| BAAAA | 228th to 243rd ranked states | 235.5 | -7.825 | -0.189 | 2 | 4.0% | 4 | 8.0% |
| A | Dead cannot be located using LOD tasks | N/A | N/A | N/A | 15 | 30.0% | 23 | 46.0% |
| Mean rescaled utility for 33333 (excluding respondents who considered 33333 to be better than dead) | | | | | -1.122 | | -1.341 | |
| Mean rescaled utility for 33333 (assuming a rescaled utility of 0 for respondents who considered 33333 to be better than dead) | | | | | -0.785 | | -0.724 | |
| Median rescaled utility for 33333 | | | | | -0.729 | | -0.189 | |

**Figure A1.** Box-plots of TTO and rescaled VAS values for health state 33333 for extended sample^a^


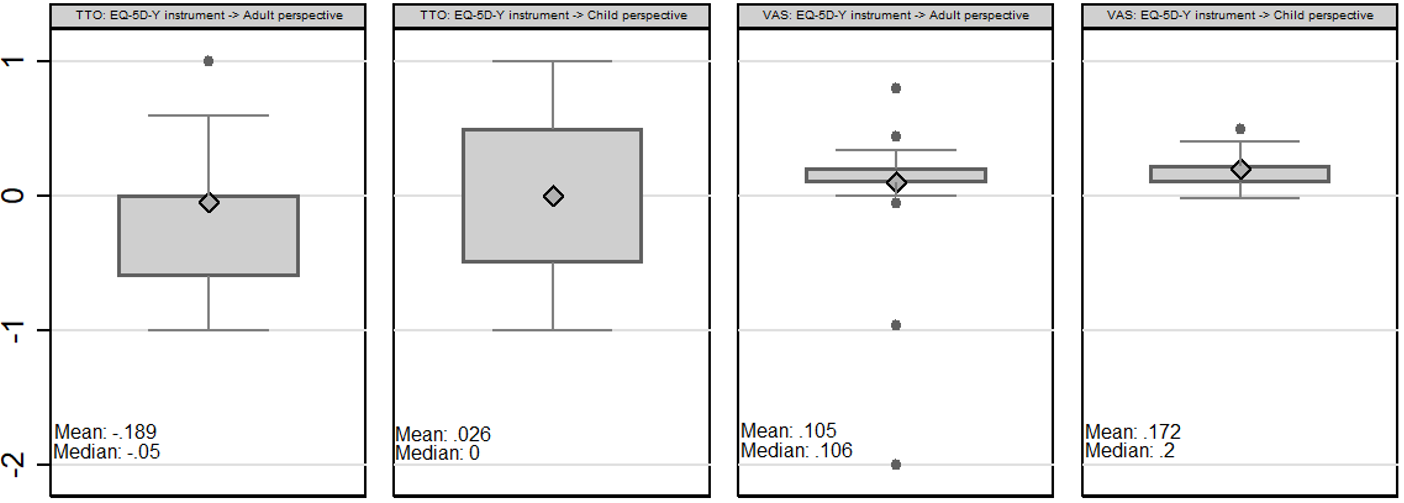


a One outlier VAS value lower than -3 was removed from the graph for scaling purposes
